# AID-expressing epithelium is protected from oncogenic transformation by an NKG2D surveillance pathway

**DOI:** 10.15252/emmm.201505348

**Published:** 2015-08-17

**Authors:** Arantxa Pérez-García, Pablo Pérez-Durán, Thomas Wossning, Isora V Sernandez, Sonia M Mur, Marta Cañamero, Francisco X Real, Almudena R Ramiro

**Affiliations:** 1 B Cell Biology Lab, Centro Nacional de Investigaciones Cardiovasculares Carlos III (CNIC)Madrid, Spain; 2 Roche Diagnostics GmbHPenzberg, Germany; 3 Epithelial Carcinogenesis Group, Spanish National Cancer Research Centre (CNIO)Madrid, Spain

**Keywords:** activation-induced deaminase, cancer, epithelium, NKG2D, pancreas

## Abstract

Activation-induced deaminase (AID) initiates secondary antibody diversification in germinal center B cells, giving rise to higher affinity antibodies through somatic hypermutation (SHM) or to isotype-switched antibodies through class switch recombination (CSR). SHM and CSR are triggered by AID-mediated deamination of cytosines in immunoglobulin genes. Importantly, AID activity in B cells is not restricted to Ig loci and can promote mutations and pro-lymphomagenic translocations, establishing a direct oncogenic mechanism for germinal center-derived neoplasias. AID is also expressed in response to inflammatory cues in epithelial cells, raising the possibility that AID mutagenic activity might drive carcinoma development. We directly tested this hypothesis by generating conditional knock-in mouse models for AID overexpression in colon and pancreas epithelium. AID overexpression alone was not sufficient to promote epithelial cell neoplasia in these tissues, in spite of displaying mutagenic and genotoxic activity. Instead, we found that heterologous AID expression in pancreas promotes the expression of NKG2D ligands, the recruitment of CD8^+^ T cells, and the induction of epithelial cell death. Our results indicate that AID oncogenic potential in epithelial cells can be neutralized by immunosurveillance protective mechanisms.

## Introduction

Activation-induced deaminase (AID) is the enzyme that initiates the reactions of secondary antibody diversification: somatic hypermutation (SHM) and class switch recombination (CSR) (Muramatsu *et al*, 2000). These reactions enable the generation of antibodies with increased affinity for antigen (SHM) and with diversified, specialized functions for antigen removal (CSR), and are therefore critical for a competent immune response (Di Noia & Neuberger, 2007; Stavnezer *et al*, 2008; Alt *et al*, 2013; Robbiani & Nussenzweig, 2013). Accordingly, defective AID activity promotes a Hyper-IgM immunodeficiency syndrome in humans (Revy *et al*, 2000). AID triggers SHM and CSR by direct deamination of cytosine nucleosides in the DNA of immunoglobulin genes, resulting in the generation of U:G mismatches (Petersen-Mahrt *et al*, 2002; Alt *et al*, 2013; Robbiani & Nussenzweig, 2013). These U:G mismatches are in turn processed by alternative repair pathways that ultimately lead in SHM to the fixation of a mutation, and in CSR to a DNA double-strand break (DSB) and a recombination reaction (Di Noia & Neuberger, 2007; Stavnezer *et al*, 2008; Alt *et al*, 2013; Robbiani & Nussenzweig, 2013).

Activation-induced deaminase activity is not confined to immunoglobulin genes and can promote mutations and DSB followed by illegitimate chromosomal translocations in other regions of the genome (Ramiro *et al*, 2004, 2006; Liu *et al*, 2008; Robbiani *et al*, 2008, 2009). Importantly, chromosomal translocations are the hallmark of mature B-cell lymphomas, the most frequent of all human lymphomas. Indeed, AID deficiency delays the onset of lymphomagenesis in the mouse (Ramiro *et al*, 2004; Kovalchuk *et al*, 2007; Pasqualucci *et al*, 2008), establishing a direct link between AID collateral genotoxic activity and neoplastic transformation in B lymphocytes.

In the last few years, it has become clear that AID expression is not, as originally thought, exclusively restricted to activated B cells. AID expression has been reported in several tissues, including gastric, hepatic, and gut epithelia (Endo *et al*, 2007, 2008; Matsumoto *et al*, 2007; reviewed in Marusawa *et al* (2011)). AID expression in these tissues is most frequently associated with inflammatory conditions and the activation of the NF-κB pathway (Endo *et al*, 2007; Matsumoto *et al*, 2007) and has been claimed to promote the accumulation of mutations in epithelial cells (Matsumoto *et al*, 2007, 2010; Takai *et al*, 2009; reviewed in Marusawa *et al* (2011)). Given that chronic inflammation in epithelial tissues predisposes to cancer development (Mantovani *et al*, 2008), the finding that the mutagenic activity of AID can be induced in an inflammatory context has fostered the idea that AID might contribute to or even constitute the link between inflammation and cancer (Takai *et al*, 2012; reviewed in Marusawa *et al* (2011)).

Several gain-of-function mouse models have been generated to address the contribution of AID to neoplastic transformation. Ubiquitous AID overexpression led mostly to early T cell neoplasia (Okazaki *et al*, 2003), hampering a thorough analysis of other malignancies. In contrast, B-cell-specific AID overexpression did not result in lymphomagenesis (Muto *et al*, 2006; Robbiani *et al*, 2009) unless the tumor suppressor p53 was removed (Robbiani *et al*, 2009). However, to date, the impact of specific AID expression in epithelial tissues, classically subject to inflammation-induced neoplastic transformation, has not been addressed. Here, we aimed to test this possibility directly by generating conditional knock-in models of AID overexpression. AID expressed in colon and pancreas epithelia was not sufficient to promote carcinogenesis, in spite of being expressed at high levels and displaying genotoxic activity. Instead, AID triggered the expression of NKGD2 ligands and the recruitment of immune cells and promoted a cytotoxic response and cell death. Our data indicate that the oncogenic potential of AID in epithelial cells is neutralized by an immunosurveillance pathway that prevents the expansion of pretumoral cells.

## Results

### Inflammation-induced AID does not contribute to carcinogenesis

**Figure 1:**
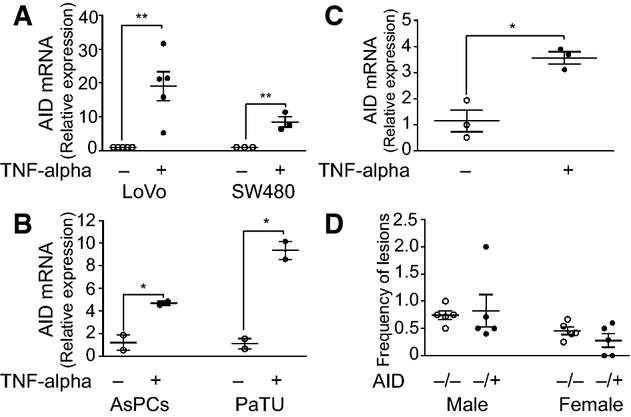
Inflammation-induced AID expression does not contribute to carcinogenesis A–C AID expression was analyzed in colon and pancreatic human cell lines and in pancreas explants from C57BL/6 mice. Samples were treated as indicated with 50 ng/ml TNF-α. (A) qRT–PCR analysis of AID expression in LoVo and SW480 colon cell lines. *n *=* *5 (LoVo); 3 (SW480). **P-value: LoVo: 0.0017; SW480: 0.0079. (B) qRT–PCR analysis of AID expression in AsPC and PaTU-8988S pancreatic cell lines (*n *=* *2). *P-value: AsPC: 0.0369; PaTU-8988S: 0.0119. (C) qRT–PCR analysis of AID expression in pancreatic explants from wild-type mice (*n *=* *3). *P =* *0.0242.
D AID^−/−^ or AID^+/−^ mice were treated with 3% DSS for 10 cycles, and colonic sections were analyzed by histologic inspection after H/E staining. Graphs represent mean frequency values of adenoma and adenocarcinoma lesions of five independent experiments. *n *=* *28 (AID^−/−^ males); 35 (females); 23 (AID^+/−^ males); 25 (females). *P-*value: male: 0.8; female: 0.246. Data information: All data are mean values ± SEM. Statistical differences were analyzed by two-tailed unpaired Student’s *t*-test.

Inflammation is known to play a critical role in the etiology of colorectal and pancreatic ductal adenocarcinoma (reviewed in Feagins *et al* (2009); Vonderheide & Bayne (2013)). To investigate whether inflammatory conditions promote AID expression in these tissues, we treated human epithelial cell lines derived from colorectal adenocarcinoma (LoVo and SW480) and pancreatic adenocarcinoma (AsPC and PaTU) with the pro-inflammatory cytokine TNF-α and measured AID expression by qRT-PCR. TNF-α stimulation increased AID mRNA expression in all cell lines analyzed (Fig 1A and B). To assess whether primary, non-transformed cells were also able to express AID in response to inflammatory stimuli, we generated explants from mouse pancreatic acinar cells and treated them with TNF-α. As with the human tumor cells, mouse primary epithelial cells expressed AID upon exposure to TNF-α (Fig 1C). TNF-α treatment typically induced 4–30-fold increases in AID mRNA levels in the different cell types tested, consistent with previous findings in liver, gastric and colorectal cell lines (Endo *et al*, 2007, 2008; Matsumoto *et al*, 2007). Together, these data confirm previous results showing that inflammatory stimuli can trigger AID expression in cell lines originated from human colorectal adenocarcinoma (Endo *et al*, 2008), and show that pancreatic adenocarcinoma cells and primary pancreatic cells are also responsive to TNF-α treatment.

Inflammation-induced AID expression has been proposed to contribute to or even be the leading cause of some epithelium-derived tumors, such as colorectal adenocarcinoma (Marusawa *et al*, 2011). To address whether endogenous AID expressed in epithelium under inflammatory conditions could contribute to carcinogenesis, we made use of the well-established model of dextran sulfate sodium (DSS)-induced colitis-associated cancer (CAC) (Cooper *et al*, 2000). AID^−/−^ mice or AID^+/−^ littermates were treated for 10 cycles with 3% DSS and evaluated by pathological criteria. We found that the frequency of oncogenic lesions was not significantly different in AID^−/−^ versus AID^+/−^ mice (Fig 1D). We conclude that endogenous AID does not significantly contribute to colorectal adenocarcinoma in the DSS-induced CAC model.

### Conditional AID expression in epithelial cells does not promote adenocarcinoma development

**Figure 2:**
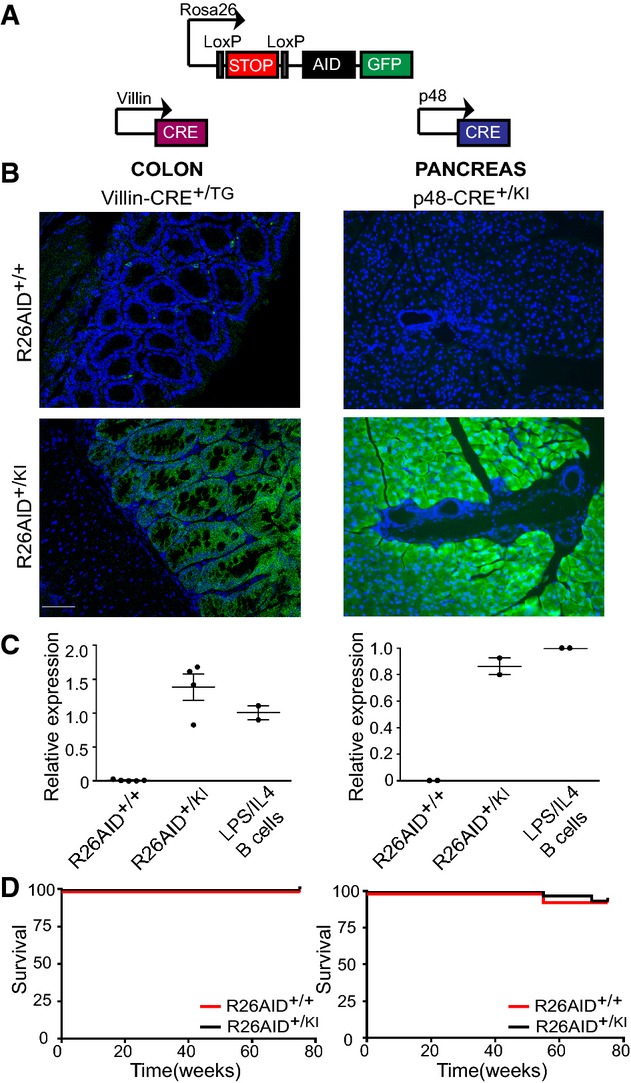
Heterologous AID expression does not promote carcinoma development Schematic of the constructs used for conditional expression of AID in epithelial cells. An AID-IRES-GFP cassette preceded by a transcriptional STOP flanked by LoxP sites was introduced by homologous recombination within the endogenous Rosa26 locus (R26AID^+/KI^ mice, top). R26AID^+/KI^ mice were bred with Villin-CRE and p48-CRE mice to achieve specific AID expression in colon and pancreas, respectively (bottom).
GFP immunofluorescence in colonic and pancreatic tissue from R26AID^+/KI^VillinCRE^+/TG^ and R26AID^+/KI^p48CRE^+/KI^ mice. Scale bar: 50 μm.
qRT–PCR analysis of AID expression in colonic and pancreatic tissue from R26AID^+/KI^VillinCRE^+/TG^ and R26AID^+/KI^p48CRE^+/KI^ mice. *n *=* *5 (R26AID^+/+^ VillinCRE^+/TG^); 4 (R26AID^+/KI^VillinCRE^+/TG^); 2 (R26AID^+/+^p48CRE^+/KI^); 2 (R26AID^+/KI^p48CRE^+/KI^). LPS+IL4-stimulated B cells are shown as a positive control (*n *=* *2). Bars show mean values ± SEM normalized to LPS+IL4-treated B cells.
Kaplan–Meier survival curves for R26AID^+/KI^VillinCRE^+/TG^ (left) (*n *=* *47 (R26AID^+/+^ VillinCRE^+/TG^); 38 (R26AID^+/KI^VillinCRE^+/TG^)) and R26AID^+/KI^ p48CRE ^+/KI^ mice (right) (*n* = 39 (R26AID^+/+^p48CRE^+/KI^); 23 (R26AID^+/KI^p48CRE^+/KI^)).

The absence of a significant contribution of endogenous AID to carcinogenesis in DSS-treated mice could be explained by an insufficient amount of AID in this model. Indeed, AID expression is known to be limiting for its activity in B cells (Sernandez *et al*, 2008), and AID levels in B cells are typically 100–1,000 fold higher than those detected in epithelial cells under inflammatory conditions (unpublished observations). To directly evaluate whether AID expression can contribute to carcinogenesis, we generated two mouse models for conditional AID expression in epithelial cells of colonic and pancreatic origin (Fig 2A). We introduced an AID-GFP-encoding cassette in the endogenous Rosa26 locus preceded by a transcriptional stop flanked by two loxP sites (R26AID^+/KI^ mice). To achieve specific expression of AID in epithelial cells, we bred R26AID^+/KI^ mice with mice expressing the Cre-recombinase under a villin promoter, which specifically drives expression in colon (el Marjou *et al*, 2004) (R26AID^+/KI^Villin-CRE^+/TG^ mice), or the pancreas-specific Ptf1 (p48) gene (Kawaguchi *et al*, 2002) (R26AID^+/KI^ p48-CRE^+/KI^ mice). R26AID^+/+^Villin-CRE^+/TG^ and R26AID^+/+^p48-CRE^+/KI^ mice were used as controls. To confirm that the Rosa26 AID-GFP cassette was functional, we first evaluated the expression of the reporter protein GFP by immunofluorescence in colon of R26AID^+/KI^Villin-CRE^+/TG^ mice and pancreas of R26AID^+/KI^p48-CRE^+/KI^ mice (Fig 2B). GFP was expressed in R26AID^+/KI^Villin-CRE^+/TG^ colon and R26AID^+/KI^p48-CRE^+/KI^ pancreas but not in control mice (Fig 2B) or in other tissues (not shown). We next measured AID transcript levels by qRT-PCR. In R26AID^+/KI^ Villin-CRE^+/TG^ and R26AID^+/KI^p48-CRE^+/KI^ mice, the amount of AID in the targeted epithelial tissues was similar to that found in B cells activated *in vitro* with LPS + IL4, whereas AID expression in control mice remained at background level (Fig 2C). AID is thus expressed in the epithelium of R26AID^+/KI^Villin-CRE^+/TG^ colon and R26AID^+/KI^p48-CRE^+/KI^ pancreas at levels known to be functional in B cells.

**Figure EV1:**
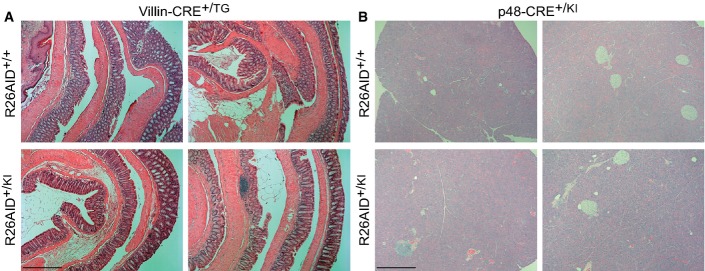
Heterologous AID expression does not promote carcinoma development Representative H/E stainings in colonic tissue from 75-week-old R26AID^+/+^VillinCRE^+/TG^ (top) and R26AID^+/KI^VillinCRE^+/TG^ (bottom) mice. Scale bar: 500 μm.
Representative H/E stainings in pancreatic tissue from 75-week-old R26AID^+/+^p48CRE^+/KI^ (top) and R26AID^+/KI^p48CRE^+/KI^ (bottom) mice. Scale bar: 500 μm.

To assess the contribution of AID to adenocarcinoma development, we monitored tumor incidence in R26AID^+/KI^Villin-CRE^+/TG^ and R26AID^+/KI^p48-CRE^+/KI^ mice. The onset of pancreatic and colorectal adenocarcinoma in a variety of mouse models ranges from 5–6 months to 1–1.5 years (Fodde & Smits, 2001; Aguilar *et al*, 2004; Martinelli *et al*, 2015). Therefore, to avoid confounding results arising from spontaneous tumorigenesis in very old mice, we set analysis end points at 75–100 weeks. Survival of R26AID^+/KI^Villin-CRE^+/TG^ mice was indistinguishable from that of R26AID^+/+^ Villin-CRE^+/TG^ littermate controls (Fig 2D, left). Likewise, survival of R26AID^+/KI^p48-CRE^+/KI^ did not differ from that of R26AID^+/+^ p48-CRE^+/KI^ controls (Fig 2D, right). To rule out the presence of early malignancies in aged animals, we performed thorough pathological analysis of colon and pancreas sections of all animals, but could not detect any tumor development in R26AID^+/KI^Villin-CRE^+/TG^ and R26AID^+/KI^p48-CRE^+/KI^ animals at 75–100 weeks (Fig EV1). Expression of AID in colon or pancreatic epithelial cells is thus not sufficient to promote tumor development.

### AID generates mutations and DNA double-strand breaks in pancreatic epithelium

**Figure 3:**
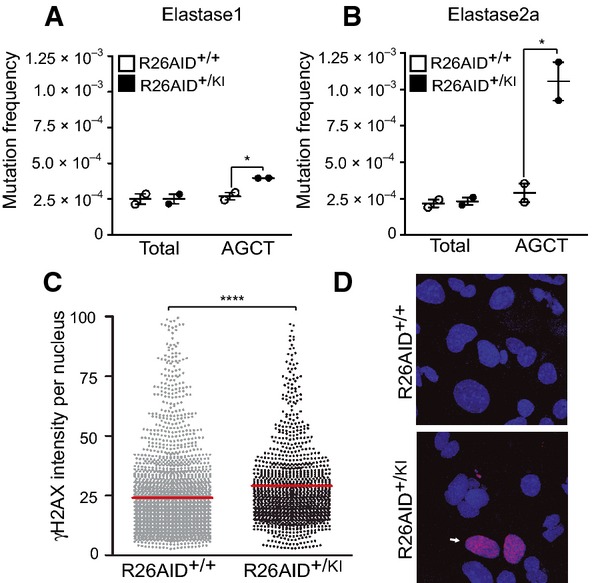
AID expression in pancreas promotes DNA lesions A, B Analysis of AID mutagenic activity in Elastase1 (A) and Elastase2a (B) by next-generation sequencing. Pancreatic DNA was isolated from pools of R26AID^+/KI^p48CRE^+/KI^ and control R26AID^+/+^p48CRE^+/KI^ 20-week-old mice, and then PCR-amplified with specific primers and sequenced as previously described (Perez-Duran *et al*, 2012). Graphs show cytosine mutation frequency overall (total) or at AGCT hotspots. *n *=* *2. *P-value: Elastase1: 0.0382; Elastase2a: 0.009.
C HTM-mediated quantification of γH2AX intensities per nuclei in pancreas explants cultured *in vitro* for 6 days. Red lines show mean values. Results of two independent experiments are shown. ****P < 0.0001.
D Representative images of γH2AX staining in pancreas explants from R26AID^+/+^p48CRE^+/KI^ (top) or R26AID^+/KI^p48CRE^+/KI^ mice (bottom) (40× magnification). White arrow points a representative positive cell. Data information: Statistical differences were analyzed by two-tailed unpaired Student’s *t*-test.

The failure of AID expression to trigger tumorigenesis prompted us to evaluate its activity in epithelial cells. We first analyzed the *in vivo* mutagenic activity of ectopically expressed AID. The primary target sequences for AID mutagenic activity are immunoglobulin genes; although other genes are known to be susceptible to AID-induced mutagenesis, this occurs at much lower rates (∼10^−4^ mutations/bp) and the mechanisms responsible for this susceptibility are poorly understood. One of the best-characterized requirements for AID activity is that the target sequence be transcriptionally active (Chaudhuri *et al*, 2003; Ramiro *et al*, 2003; Pavri & Nussenzweig, 2011). To simplify the mutagenesis analysis, we made use of the p48 pancreatic AID expression model to take advantage of the known low complexity transcriptome of acinar cells (MacDonald *et al*, 2010). AID preferentially targets the consensus hotspots WRCY/RGYW and particularly AGCT motifs (Rogozin & Kolchanov, 1992; Pham *et al*, 2003; Perez-Duran *et al*, 2012). Based on this, we analyzed the presence of mutations in 800–900 bp downstream of the transcriptional start site of two highly transcribed genes in pancreas, Elastase1 (Ela1) and Elastase2 (Ela2a), by next-generation sequencing, which allows large number of mutations to be analyzed at a very high depth (Perez-Duran *et al*, 2012). This analysis revealed that mutations are specifically accumulated at the Ela1 and Ela2 genes in R26AID^+/KI^p48-CRE^+/KI^ animals (Fig 3A and B) at frequencies similar to those of other non-Ig genes in B cells (Liu *et al*, 2008). AID activity was verified for Ela1 by conventional Sanger sequencing (Table 1). In contrast to previous reports (Matsumoto *et al*, 2007), we did not detect AID-induced mutations at the tumor suppressor gene Trp53, where mutation frequency was identical in R26AID^+/KI^p48-CRE^+/KI^ mice and R26AID^+/+^p48-CRE^+/KI^ controls (Table 1).

**Table 1:** Analysis of AID mutagenic activity by Sanger sequencing

	Genotype	Total clones analyzed	Mutations	Total bp sequenced	Frequency (×10^4^)
Elastase1	R26AID^+/+^ p48-CRE^+/KI^	84	4	70,018	0.571
	R26AID^+/KI^ p48-CRE^+/KI^	82	13	69,355	1.87
Trp53	R26AID^+/+^ p48-CRE^+/KI^	66	0	59,472	0
	R26AID^+/KI^ p48-CRE^+/KI^	59	1	53,936	0.185

To assess whether AID activity in R26AID^+/KI^p48-CRE^+/KI^ mice leads not only to mutations but also to more aggressive lesions, such as DSBs, we quantified γ-H2AX, a histone phosphorylation produced in response to this type of DNA damage. For this analysis, we generated acinar-cell explants from R26AID^+/KI^p48-CRE^+/KI^ and control mice, stained them with anti-γH2AX, and quantified the intensity of staining per nucleus by high-throughput microscopy (HTM). We found that AID expression in R26AID^+/KI^p48-CRE^+/KI^ mice promoted a significant increase in the levels of γ-H2AX (Fig 3C and D), indicating that AID generates DSBs in this cellular context.

### AID induces NKG2D ligands, T cell recruitment and apoptotic cell death in pancreas

**Figure 4:**
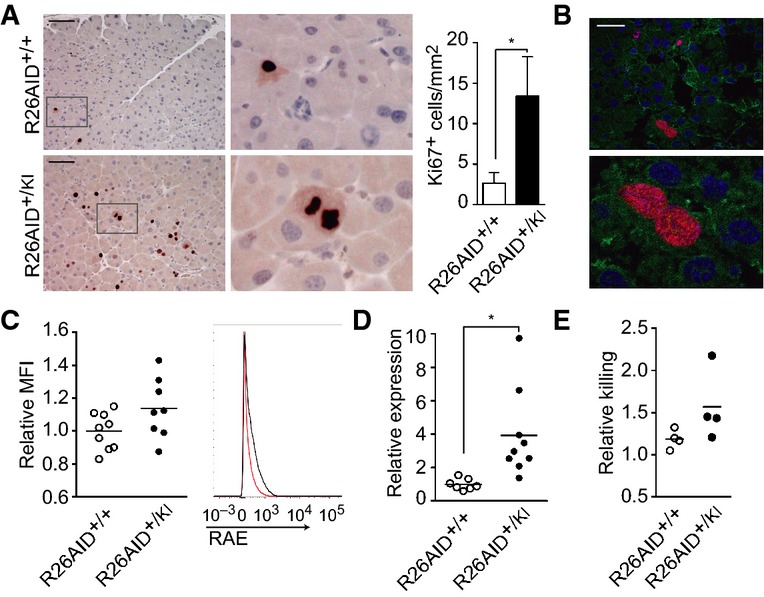
AID expression in pancreas promotes proliferation and NKG2D ligand expression Representative images of Ki67 staining in pancreas from aged (75-week-old) R26AID^+/+^p48CRE^+/KI^ (top) or R26AID^+/KI^p48CRE^+/KI^ mice (bottom). Detail is shown on the right. Scale bar: 100 μm. Graph shows quantification of Ki67-positive epithelial cells per mm^2^ of tissue (*n *=* *8). *P =* *0.0266.
Representative immunofluorescence staining of 20-week-old R26AID^+/KI^p48CRE^+/KI^ mice: blue, DAPI; red, Ki67; green, CK8. Scale bar: 50 μm. Detail is shown on the bottom.
Quantitative FACS analysis of RAE expression in pancreatic explants from R26AID^+/+^p48CRE^+/KI^ and R26AID^+/KI^p48CRE^+/KI^ mice. Left: Graph shows mean fluorescence intensity. Each dot represents an individual mouse. *n *=* *9 (R26AID^+/+^p48CRE^+/KI^); 8 (R26AID^+/KI^p48CRE^+/KI^). *P *=* *0.077. Right: Representative FACS staining for RAE in explants from R26AID^+/+^p48CRE^+/KI^ (red) and R26AID^+/KI^p48CRE^+/KI^ mice (black).
Analysis of RAE expression by ddPCR in aged (75-week-old) mice. Data are presented as the percentage of positive drops normalized to the mean control value. Each point represents an individual mouse and shows the mean amplification from two independent experiments *n *=* *7 (R26AID^+/+^p48CRE^+/KI^); 9 (R26AID^+/KI^p48CRE^+/KI^). *P =* *0.012.
Analysis of killing activity. Primary explants of pancreatic cells from R26AID^+/+^p48CRE^+/KI^ or R26AID^+/KI^p48CRE^+/KI^ mice were cultured with primary NK cells, and killing activity was assessed as described in Materials and Methods (*n *=* *4). *P *=* *0.19. Data information: Statistical differences were analyzed by two-tailed unpaired Student’s *t*-test.

**Figure EV2:**
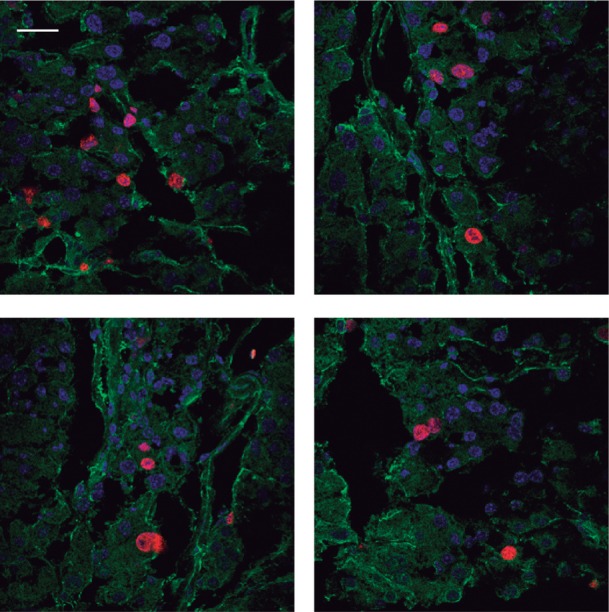
Ki67 is expressed in pancreatic epithelial cells Representative immunofluorescence stainings of pancreatic tissue of 20-week-old R26AID^+/KI^p48CRE^+/KI^ mice: blue, DAPI; red, Ki67; green, CK8. Scale bar: 50 μm

Activation of the DNA damage response (DDR) pathway induces the expression of NKG2D ligands in epithelial cells, which are in turn recognized by NKG2D receptors expressed by NK cells and subsets of T cells (Diefenbach *et al*, 2001; Gasser *et al*, 2005; Champsaur & Lanier, 2010; Raulet *et al*, 2013). This cross talk promotes the elimination of precancerous cells and is therefore a mechanism to prevent tumor development (Guerra *et al*, 2008). Given that AID expression in pancreas promotes mutations and DNA damage without leading to tumor development, we sought for the evidence of precancerous cells and found that pancreas from aged R26AID^+/KI^p48-CRE^+/KI^ mice contained more proliferating cells, as assessed by Ki67 staining, than control pancreas (Fig 4A), indicating that pancreatic AID expression leads to an abnormal rate of cell division. The epithelial identity of Ki67^+^ cells was confirmed both by morphology (Fig 4A, magnified micrograph on the right) and by staining with the epithelium-specific anti-cytokeratin 8 antibody (Figs 4B and EV2). We next asked whether the NKG2D immune surveillance pathway could be in play in R26AID^+/KI^p48-CRE^+/KI^ mice. To test this hypothesis, we first analyzed the expression of the Raeε NKG2D ligand in epithelial cells from pancreatic explants of R26AID^+/KI^p48-CRE^+/KI^ and control mice by flow cytometry. Acinar cells from R26AID^+/KI^p48-CRE^+/KI^ mice expressed higher levels of Raeε than their control littermates, although the difference was not statistically significant (Fig 4C). To assess whether RAE ligands were expressed by pancreatic cells *in vivo*, we prepared pancreas extracts from aged (75-week-old) R26AID^+/KI^p48-CRE^+/KI^ mice and controls and measured the amount of five RAE isoforms by droplet digital PCR (ddPCR). With this technique, each sample is fractioned into thousands of droplets, in which PCR amplification reactions occur independently, thereby increasing the sensitivity and quantitative potential of the amplification. Amplification of RAE isoforms was detected in more drops from R26AID^+/KI^p48-CRE^+/KI^ samples than from controls (Fig 4D), indicating that AID promotes the expression of NKG2D ligands in pancreas, most likely as a result of DSB and DDR. We found that primary explants from R26AID^+/KI^p48-CRE^+/KI^ tended to be more sensitive to NK-mediated killing than R26AID^+/+^p48-CRE^+/KI^ littermate controls (Fig 4E), indicating that NKG2D ligand expression in AID-expressing pancreas is functional.

**Figure 5:**
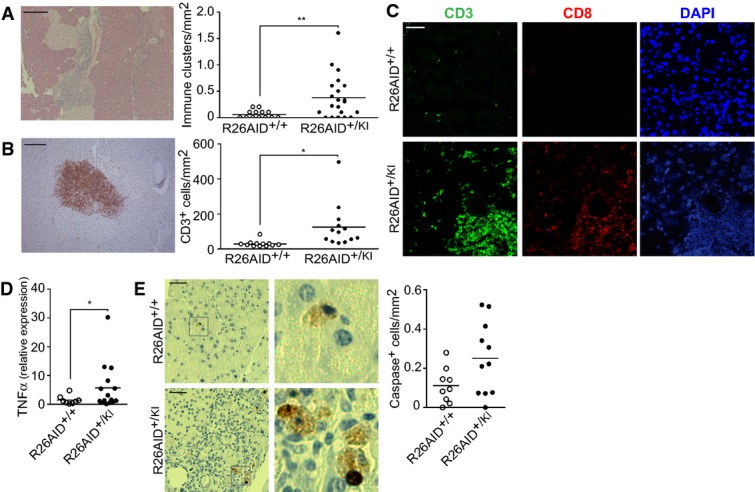
AID expression in pancreas promotes immune infiltration and cell death Hematoxylin–eosin (HE) staining of pancreas from aged (75-week-old) R26AID^+/+^p48CRE^+/KI^ and R26AID^+/KI^p48CRE^+/KI^ mice. Left: Representative HE staining showing an immune infiltrate in a R26AID^+/KI^p48CRE^+/KI^ mouse. Scale bar: 200 μm. Right: Quantification of number of foci per mm^2^ of tissue. *n *=* *13 (R26AID^+/+^p48CRE^+/KI^); 21 (R26AID^+/KI^p48CRE^+/KI^). **P =* *0.0098.
CD3 immunohistochemistry of pancreas from 75-week-old R26AID^+/+^p48CRE^+/KI^ and R26AID^+/KI^p48CRE^+/KI^ mice. Left: Representative image of a CD3 infiltrate in a R26AID^+/KI^p48CRE^+/KI^ mouse. Scale bar: 100 μm. Right: Quantification of the number of CD3-positive cells per mm^2^ of tissue. *n *=* *12 (R26AID^+/+^p48CRE^+/KI^); 13 (R26AID^+/KI^p48CRE^+/KI^). *P =* *0.0149.
Representative immunofluorescence staining of CD3 and CD8 in pancreas from 20-week-old R26AID^+/+^p48CRE^+/KI^ and R26AID^+/KI^p48CRE^+/KI^ mice. Scale bar: 20 μm.
TNF-α expression. Total RNA was isolated from pancreas of aged (75-week-old) R26AID^+/KI^p48CRE^+/KI^ and control mice, and TNF-α expression was quantified by qRT-PCR. Each dot represents an individual mouse. *n *=* *8 (R26AID^+/+^p48CRE^+/KI^); 15 (R26AID^+/KI^p48CRE^+/KI^). *P =* *0.0392.
Cell death detection. Pancreas from 75-week-old R26AID^+/+^p48CRE^+/KI^ and R26AID^+/KI^p48CRE^+/KI^ mice were stained with anti-caspase-3. Left: Representative staining from an R26AID^+/+^p48CRE^+/KI^ mouse (top) and an R26AID^+/KI^p48CRE^+/KI^ mouse (bottom). Scale bar: 50 μm. Right: Quantification of number of cells per mm^2^ of tissue. Each dot represents an individual mouse. *n *=* *9 (R26AID^+/+^p48CRE^+/KI^); 11 (R26AID^+/KI^p48CRE^+/KI^). *P *=* *0.0545. Data information: Statistical differences were analyzed by two-tailed unpaired Student’s *t*-test.

We next asked whether the expression of RAE ligands promoted the recruitment of immune cells to R26AID^+/KI^p48-CRE^+/KI^ pancreas *in vivo*. Hematoxylin–eosin staining of pancreas sections from aged mice clearly revealed the presence of immune infiltrates in AID-expressing pancreas of R26AID^+/KI^p48-CRE^+/KI^ mice (Fig 5A). The composition of these immune infiltrates was analyzed by antibody staining to detect macrophages (F4/80), B cells (Pax5) and T cells (CD3). The vast majority of cells in the immune infiltrates of R26AID^+/KI^p48-CRE^+/KI^ mice were CD3^+^ T cells (Fig 5B), with only a negligible contribution from B cells and macrophages (not shown). To discount age-related effects, we analyzed 20-week-old mice, finding that the accumulation of T cell infiltrates is detectable in these young animals (Fig 5C). The main NKG2D-expressing T cell subset is the CD8^+^ population (Raulet *et al*, 2013), and immunofluorescence analysis of immune infiltrates revealed that a high proportion of the CD3^+^ infiltrate is composed of CD8^+^ T cells (Fig 5C), a finding consistent with the reported recruitment of CTL cells to pancreatic islets transgenically expressing Raeε (Markiewicz *et al*, 2012). Finally, we found that aged R26AID^+/KI^p48-CRE^+/KI^ mice had significantly higher levels of pancreatic TNF-α mRNA than control littermates (Fig 5D), indicating that AID promotes the expression of effector cytotoxicity. Consistently, R26AID^+/KI^p48-CRE^+/KI^ pancreas contained cells undergoing apoptotic cell death, detected by caspase-3 immunohistochemistry (Fig 5E, *P* = 0.054). Together, these results indicate that heterologous AID expression in pancreas promotes a cytotoxic response, most likely arising from the generation of genotoxic activity and NKG2D ligand expression and the recruitment of NKG2D-expressing CTL cells.

## Discussion

In recent years, the finding that inflammatory cues induce AID expression in epithelial cells has boosted interest in the notion that AID might promote carcinogenesis and even be the causative link between inflammation and neoplastic transformation (Marusawa *et al*, 2011; Takai *et al*, 2012). In this regard, our analyses of AID expression in response to TNF-α have confirmed previous data in colonic cell lines and have expanded these observations to pancreatic cell lines. In addition, we found that primary pancreatic epithelium is also sensitive to TNF-α, suggesting that AID expression can indeed take place *in vivo* in pro-inflammatory contexts.

We wanted to further explore the physiological relevance of AID expression in promoting epithelium malignant transformation. We found that AID deficiency does not reduce the incidence of oncogenic lesions in an inflammation-induced carcinoma model. Our results contrast with the finding that AID deficiency reduces colon carcinogenesis in IL10^−/−^ mice (Takai *et al*, 2012). It may be that the effect reported by Takai *et al* is not specifically driven by epithelial cells, but rather by B cells in IL10^−/−^ mice, a possibility that could be tested using conditionally rather than constitutively AID-deficient animals.

Previous reports have claimed that AID heterologous expression leads to epithelial cell neoplasia in various tissues (Endo *et al*, 2008; Takai *et al*, 2009, 2012; Marusawa *et al*, 2011). In those studies, overexpression was always achieved with transgenes, none of which was epithelium specific, and the incidence of neoplasias was extremely low, and varied with the insertion sites and with ongoing mouse generations (Okazaki *et al*, 2003). In contrast, here we have developed epithelium-specific conditional knock-in models, thus avoiding both widespread expression and transgene-derived artifacts. Remarkably, our models allowed B-cell-like AID expression levels, a basic prerequisite for AID to be competent, given that its levels are rate limiting in its native context (Sernandez *et al*, 2008; Takizawa *et al*, 2008). We found that AID expressed ectopically in pancreatic cells is able to mutate non-immunoglobulin genes and to generate genotoxic DSBs, suggesting that in this context, there are no obvious mechanisms for negative regulation of AID activity, as they were previously proposed for transgenic AID expression in B cells (Muto *et al*, 2006). AID mutagenic activity was detected in two highly expressed pancreatic genes (Ela1 and Ela2), which is expected from the well-established link between AID activity and transcription of its target genes (Chaudhuri *et al*, 2003; Ramiro *et al*, 2003; MacDonald *et al*, 2010; Pavri & Nussenzweig, 2011).

Our data indicate that AID activity in pancreas does not promote pancreatic carcinogenesis; instead, it triggers an NKG2D-mediated cytotoxic response that would eliminate pretumoral cells and prevent carcinoma development, in line with the published finding that AID promotes an NKG2D immune response in B cells infected with the Abelson murine leukemia virus (Gourzi *et al*, 2006). The lymphomagenic potential of AID was previously shown to be dampened in B cells (Muto *et al*, 2006; Robbiani *et al*, 2009), where p53 exerts a cell-intrinsic tumor suppressor function (Robbiani *et al*, 2009). Here, we provide evidence of a further protective mechanism triggered by AID activity and carried out through an extrinsic immunosurveillance pathway in epithelial tissues. These data highlight the diversity of safeguarding events in AID-expressing cells and encourage a refined view of the previously acknowledged contribution of endogenous AID to epithelial-derived tumors.

## Materials and Methods

### Mice

R26AID mice were generated by insertion of a construct encoding mouse AID cDNA into the Rosa26 IRES-GFP targeting vector (Nyabi *et al*, 2009). AID cDNA was PCR-amplified from C57BL/6 mice (primers: forward 5′-TTCTGTGAAGACCGCAAGGCT-3′; reverse 5′-CCCTTCCCAGGCTTTGAAA-3′), cloned into the pENTR/D-TOPO Gateway vector (Invitrogen), and subsequently recombined into the Rosa 26 targeting vector, in which the cloned construct is preceded by a loxP-flanked transcriptional stop cassette and followed by an internal ribosomal entry site and GFP. The construct was linearized with PvuI before electroporation into hybrid 129/C57BL/6 ES cells. Clones positive for homologous recombination in the Rosa26 locus were identified by Southern blot of EcoRV-digested genomic DNA hybridized with a 5′-arm Rosa26 probe. R26AID mice were backcrossed to C57BL/6 background for 5 generations. R26AID mice were crossed with Villin-CRE^+/KI^ mice (el Marjou *et al*, 2004) and p48-CRE^+/KI^ mice (Kawaguchi *et al*, 2002) to promote expression of AID in colonic and pancreatic epithelial cells, respectively. Both Villin-CRE and p48-CRE mice were backcrossed to C57BL/6 for 5 generations. Balb/c AID^−/−^ mice were generated by backcrossing AID^−/−^ mice (Muramatsu *et al*, 2000) for 6 generations by speed congenics (Ramiro *et al*, 2004). Mice of both genders were used unless specified otherwise. All animals were housed in the Centro Nacional de Investigaciones Cardiovasculares animal facility under a 12-h light/dark cycle with food *ad libitum*.

Number of animals per group to detect biologically significant effect sizes was calculated using appropriate statistical sample size formula and indicated in the biometrical planning section of the animal license submitted to the governing authority. Blinding and randomization was not applicable to the animal studies. All animal procedures conformed to EU Directive 2010/63EU and Recommendation 2007/526/EC regarding the protection of animals used for experimental and other scientific purposes, enforced in Spanish law under RD 53/2013.

### Cell lines and primary acinar cell culture

Primary pancreatic acinar cells were isolated and cultured as described in Gout *et al* (2013). Briefly, complete pancreas from 8-week-old mice was mechanically and enzymatically digested with collagenase to obtain isolated acinar structures. Acini were grown in Waymouth’s medium supplemented with 2.5% FBS, 10 mM HEPES, 0.25 mg/ml trypsin inhibitor (Sigma) and 25 ng/ml of recombinant human epidermal growth factor (Sigma). PaTU-8988S, AsPC-1, LoVo and SW480 cells were grown in DMEM supplemented with 10% FCS and 10 mM HEPES. PaTU-8988S cell line was kindly provided by Dr Thomas Gress (University of Marburg). AsPC-1, LoVo and SW480 cell lines were obtained from the ATCC. All of them were mycoplasma negative. TNF-α (50 ng/ml) was added when indicated.

### DSS-induced colitis-associated cancer (CAC) experiments

8–10-week-old Balb/c AID^+/−^ and AID^−/−^ (Ramiro *et al*, 2004) mice were given 3% dextran sulfate sodium salt (DSS, Sigma) in their drinking water for 5 days followed by regular drinking water for 10 days. Colon samples were obtained from these mice after 10 cycles of DSS treatment, and Swiss roll preparations were fixed, embedded in paraffin for section, and stained with hematoxylin/eosin (H/E).

### Immunofluorescence

Pancreas and colon specimens were fixed with 4% paraformaldehyde, incubated with 30% sucrose, embedded in OCT compound (Olympus), and frozen in dry ice. 10-μM sections were permeabilized and blocked with Image-It FX signal enhancer (Invitrogen, Molecular Probes). The following antibodies were used: rabbit anti-GFP (Abcam, 1/100), rat anti-mouse CD8a (BD Pharmingen, 1/100), mouse anti-Cytokeratin 8 (TROMA I, 1/50), goat anti-rabbit Alexa Fluor 488 (Molecular Probes, 1/500), goat anti-mouse Alexa Fluor 488 (Molecular Probes, 1/500), and goat anti-rat Cy3 (Jackson ImmunoResearch, 1/500). Slides were mounted with Vectashield mount medium containing DAPI (Vector Laboratories).

### Immunohistochemistry

Pancreases were fixed in neutral-buffered 10% formalin solution (Sigma), embedded in paraffin blocks, and cut in 5-μM sections. H/E staining was performed using standard protocols. For immunohistochemistry, sodium citrate buffer was used for antigen retrieval. The following antibodies were used: polyclonal rabbit anti-human CD3 (Dako, 1/200), rabbit anti-Ki67 (Abcam, 1/100), and biotinylated goat anti-rabbit (Abcam, 1/200). Biotinylated antibody was detected with the ABC system using diaminobenzidine as substrate (Vector Laboratories). Images were acquired with a Leica DM2500 microscope fitted with a 20× magnification lens. Reactive cells from 10 microscope fields per pancreas were counted using ImageJ. Results are shown as the number of reactive cells per mm^2^ of tissue.

### qRT–PCR

RNA was extracted from colon samples with TRIzol (Sigma). RNA from pancreas samples was extracted with GTC solution, following a standard phenol–chloroform protocol (reagents from Sigma), followed by DNAse treatment (Qiagen). cDNA was synthesized using Random Hexamers (Roche) and SuperScript II reverse transcriptase. mRNA was quantified by SYBR green assay (Applied Biosystems), with normalization to GAPDH. The following primers were used: human-AID (forward) 5′-AAA TGT CCG CTG GGC TAA GG-3′, (reverse) 5′-GGA GGA AGA GCA ATT CCA CGT-3′; human-GAPDH (forward) 5′-GAA GGT GAA GGT CGG AGT C-3′, (reverse) 5′-GAA GAT GGT GAT GGG ATT TC-3′; mouse AID (forward) 5′-ACC TTC GCA ACA AGT CTG GCT-3′, (reverse) 5′-AGC CTT GCG GTC TTC ACA GAA-3′; mouse-GAPDH (forward) 5′-TGA AGC AGG CAT CTG AGG G-3′, (reverse) 5′-CGA AGG TGG AAG AGT GGG AG-3′; mouse-TNF-α (forward) 5′-AGC CCA CGT CGT AGC AAA CCA-3′, (reverse) 5′-ACA ACC CA CGG CTG GCA CC-3′.

### Next-generation sequencing for detection of mutations

DNA from three R26AID^+/+^p48-CRE^+/KI^ and three R26AID^+/KI^ p48-CRE^+/KI^ mice was extracted and amplified using the following oligonucleotides: Elastase1 (forward) 5′-GCA CAG CAT CTT TTG TTT GGG TAA-3′, (reverse) 5′-GGG GAC AGT GGT CTA CTC TCT-3′; Elastase2a (forward) 5′-ACG AGT CCA GGA CAA TCA GAG A-3′, (reverse) 5′-TGA TAA GGC CAC TCA TAA AAA GGA-3′. Amplification reactions were carried out with 2.5 U of Pfu Ultra (Stratagene) in a 50-μl reaction with the following profile: 94°C for 5 min followed by 25 cycles at 94°C for 10 s, 60°C for 30 s, and 72°C for 1 min. For NGS sequencing, the PCR product from two reactions per mouse were pooled, and an equimolar amount of DNA from each of the two mouse genotypes was pooled and mixed.

### γ-H2AX staining and Opera acquisition

Primary pancreatic acinar cells were grown on 96-well plates (Perkin Elmer), and γ-H2AX (Millipore, 1/500) immunofluorescence was performed using standard procedures. Images were automatically acquired from each well with the Opera High-Content Screening System (Perkin Elmer). A 40× magnification lens was used, and pictures were taken at nonsaturating conditions. Images were segmented using DAPI staining to generate masks matching cell nuclei, and the mean per-cell γ-H2AX signal was calculated.

### Droplet digital PCR

Each 20 μl ddPCR contained 10 μl of 2× ddPCR Supermix (Bio-Rad), 1 μl of cDNA generated from 1.4 μg of RNA, and 500 nM of each primer: pan-RAE (forward) 5′-TGG ACA CTC ACA AGA CCA ATG-3′, (reverse) 5′-CCC AGG TGG CAC TAG GAG T-3′; and 250 nM Taqman probe (panRAE-FAM 5′-CCA TGA TTT ATC CGC AAA GCC AGG GCC-3′). After droplet generation, a mean of 12,000 droplets was obtained per sample.

Samples were transferred into a 96-well plate (Eppendorf) and cycled in a thermal cycler (Bio-Rad) under the following conditions: 95°C for 10 min followed by 38 cycles of 94°C for 30 s, 58°C for 1 min, and a final step at 98°C for 10 min. After amplification, samples were transferred to a droplet reader (QX100 Droplet Digital PCR, Bio-Rad) from which positive-drop data were extracted with Quantasoft software. Results are represented as the proportion of positive drops in duplicates of each sample.

### NK killing assay *in vitro*

Primary pancreatic acinar cells from R26AID^+/+^p48-CRE^+/KI^ and R26AID^+/KI^p48-CRE^+/KI^ mice were isolated as described above. After 6 days of culture, acinar cells were trypsinized and stained with CFSE. NK cells were isolated by cell sorting from wild-type C57BL/6 mice. Both male and female aged 6–8 weeks were used as NK cells donors. NK cells and acinar pancreatic cells were co-cultured for 4 h in the presence of IL2 (2,000 U/ml, Peprotech) at a 1:10 (target cell:NK effector cell) ratio. Killing was analyzed by staining with DAPI by flow cytometry. Data are presented as the proportion of CFSE^+^DAPI^+^ cells normalized to the same population in cultures lacking NK cells.

### Statistics

Statistical analyses were performed with GraphPad Prism (version 6.01 for Windows, GraphPad Software, San Diego, CA, USA) using two-tailed Student’s *t*-test. *P* ≤ 0.05 was considered statistically significant. Error bars in figures represent standard error of the mean (SEM). Normal distribution of data was assessed by applying a D’Agostino & Pearson omnibus normality test. F-test was used to compare variances between groups. For the survival analyses, GraphPad Prism was used and the Mantel–Cox test was applied. Differences were considered statistically significant at *P* ≤ 0.05.

## Supporting Information

Expanded View Figures PDF

Review Process File
