# Supplementary material for: AID-expressing epithelium is protected from oncogenic transformation by an NKG2D surveillance pathway
Source: EMBO Mol Med. 2015 Aug 17;7(10):1327–36. doi: 10.15252/emmm.201505348 (PMC4604686; doi:10.15252/emmm.201505348)
Supplement: Supplementary file 1 [file emmm0007-1327-sd1.pdf]

Expanded View Figures

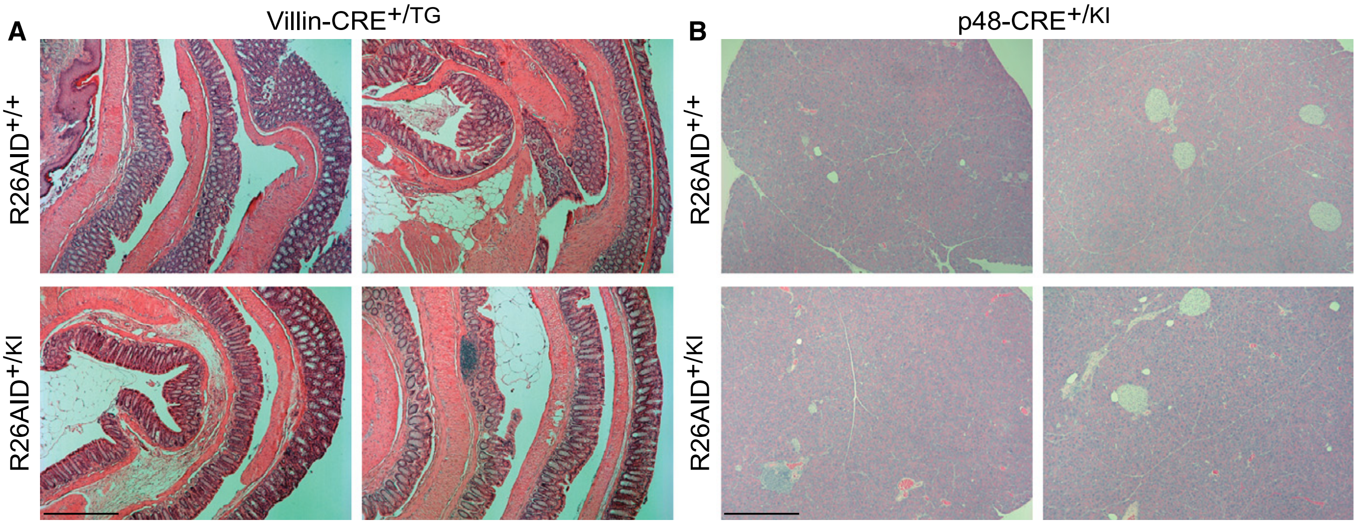

Figure EV1. Heterologous AID expression does not promote carcinoma development.
A Representative H/E stainings in colonic tissue from 75-week-old R26AID+/+VillinCRE+/TG (top) and R26AID+/KIVillinCRE+/TG (bottom) mice. Scale bar: 500 μm.
B Representative H/E stainings in pancreatic tissue from 75-week-old R26AID+/+p48CRE+/KI (top) and R26AID+/KIp48CRE+/KI (bottom) mice. Scale bar: 500 μm.

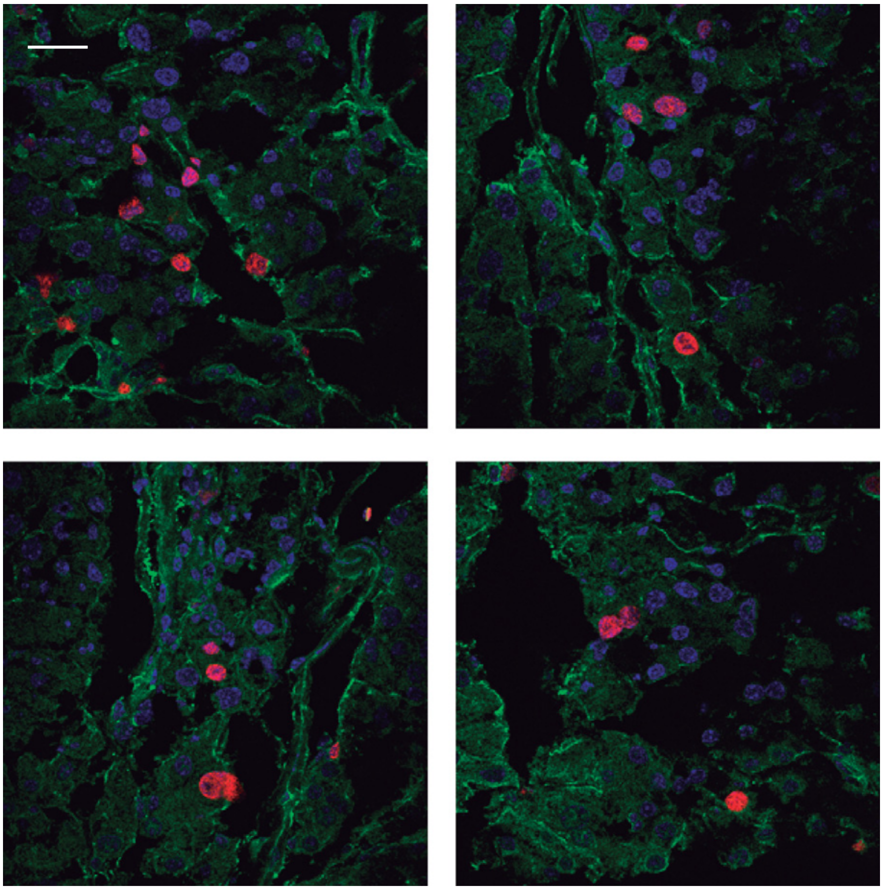

Figure EV2. Ki67 is expressed in pancreatic epithelial cells.
Representative immunofluorescence stainings of pancreatic tissue of 20-week-old R26AID+/KIp48CRE+/KI mice: blue, DAPI; red, Ki67; green, CK8. Scale bar: 50 μm

© 2015 The Authors
