# Supplementary material for: AID-expressing epithelium is protected from oncogenic transformation by an NKG2D surveillance pathway
Source: EMBO Mol Med. 2015 Aug 17;7(10):1327–36. doi: 10.15252/emmm.201505348 (PMC4604686; doi:10.15252/emmm.201505348)
Supplement: Supplementary file 2 [file emmm0007-1327-sd2.pdf]

AID-expressing epithelium is protected from oncogenic transformation by an NKG2D surveillance pathway

Arantxa Pérez-García, Pablo Pérez-Durán, Thomas Wossning, Isora Sernandez, Sonia Mur, Marta Cañamero, Francisco X. Real and Almudena R. Ramiro

Corresponding author: Almudena R. Ramiro, Centro Nacional de Investigaciones Cardiovasculares

Review timeline:

Submission date:	16 April 2015
Editorial Decision:	02 June 2015
Revision received:	23 June 2015
Editorial Decision:	08 July 2015
Revision received:	16 July 2015
Accepted:	20 July 2015

Transaction Report:

(Note: With the exception of the correction of typographical or spelling errors that could be a source of ambiguity, letters and reports are not edited. The original formatting of letters and referee reports may not be reflected in this compilation.)

Editor: Céline Carret

1st Editorial Decision 02 June 2015

Thank you for the submission of your manuscript to EMBO Molecular Medicine. We are very sorry that it has taken much longer than usual to get back to you on your manuscript. In this case we experienced significant difficulties in securing three expert and willing Reviewers. Further to this, one evaluation was delivered with considerable delay.

We have now heard back from the three referees whom we asked to evaluate your manuscript. You will see that while referees 1 and 2 are enthusiastic about the findings referee 3 is much more critical. We would like to ask you to focus on addressing the concerns of referees #1, #2 and points 1, 2 and 6 from referee #3. These issues relate to better descriptions, explanations and consistency between experiments. In addition, if you have data on hand addressing the concerns of referee #3 points 3, 4, 5, we would strongly encourage you to include these in the manuscript but we will not consider experimental additional data of these issues mandatory for further evaluation of the article.

Given these evaluations, I would like to give you the opportunity to revise your manuscript, with the understanding that the referees' concerns must be fully addressed and that acceptance of the manuscript would entail a second round of review. Please note that it is EMBO Molecular Medicine policy to allow only a single round of revision and that, as acceptance or rejection of the manuscript will depend on another round of review, your responses should be as complete as possible.

EMBO Molecular Medicine has a "scooping protection" policy, whereby similar findings that are published by others during review or revision are not a criterion for rejection. Should you decide to submit a revised version, I do ask that you get in touch after three months if you have not completed it, to update us on the status.

Please also contact us as soon as possible if similar work is published elsewhere. If other work is published we may not be able to extend the revision period beyond three months.

I look forward to receiving your revised manuscript.

***** Reviewer's comments *****

Referee #1 (Comments on Novelty/Model System):

For details please see my review below.

Referee #1 (Remarks):

The question whether inflammation-induced AID expression plays a role in carcinogenesis has not been addressed before and is of high relevance in the field. Here, the authors developed tissue specific overexpression models to determine if AID overexpression is sufficient to drive tumor development upon. The data show that AID-deficiency is not associated with a lower incidence of oncogenic lesions in a model of induced-colitis. At the same time tissue specific AID overexpression to levels typically observed in B cells was not sufficient to drive tumor development in colon and pancreas independent of the accumulation of mutations and presence of double-strand breaks. Using the pancreas model, the authors provide mechanistic evidence that activation of the DNA damage response may lead to NKG2D ligand expression and recruitment of cytotoxic T-cells to mediate cell death.

The manuscript is technically very solid in that the experiments are well controlled. The use of tissue-specific models advances significantly over the current literature and explains differences compared to previous reports of transgenic mice. The manuscript is well written and the conclusions are supported be the findings, which are novel and demonstrate that more thorough experiments are needed to define the importance of AID in epithelial tumor development.

Referee #2 (Comments on Novelty/Model System):

Although statistical analysis is missing in some places, indicated in comments to the author, the experiments utilize state of the art technology and are extremely well performed. A novel system is used to express AID conditionally in two different tissues, so that the question of AID-induced tumorogenesis can be properly addressed.

Referee #2 (Remarks):

Activation induced cytidine deaminase (AID) induces DNA mutations and breaks in B cells that promote immunoglobulin class switch recombination and somatic hypermutation, and its activities are also strongly linked to lymphomogenesis. AID expression was initially thought to be restricted to B cells, but is now found in a broader array of tissues. The biological significance and consequences of AID expression in non-B cells is not known. Perez-Garcia et al ask whether the mutagenic activities of AID do, in fact, lead to tumorogenesis in non-B cells, using a model where AID expression is known to be induced during inflammation by TNFα in endothelial cells. This model is appropriate and of interest considering the known association between the inflammatory milieu and tumorogenesis in epithelial cells, and previous reports that suggest an association between AID expression and colorectal adenocarcinoma. The manuscript is very well written, interesting, and will appeal to a broad audience.

The authors confirm that endogenous AID is induced by inflammatory stimuli when pancreatic explants are treated with TNFα. They then use an established model of DSS-induced colitis associated cancer, but find no difference in pathology between AID-deficient and AID-heterozygous mice, and conclude that endogenous AID levels do not contribute significantly to colorectal adenocarcinoma. They go on to express AID at higher levels, similar to those seen in B cells,

conditionally, under the control of promoters that direct expression specifically in colon or pancreas. This is in contrast to previous studies that have asked this question using transgenic mouse models where AID is broadly expressed and could induce secondary effects. In both tissues, they find that AID expression neither promotes tumor formation, nor impacts lifespan. Although this is essentially a negative result, it represents an important answer to a biologically important question, and the experiments were very well done so as to generate a meaningful result. Importantly, the authors go on to show that AID does in fact generate DNA lesions in these cells, and they identify a DNA damage response pathway induced in these cells that results in apoptotic death of damaged cells. Identification of this protective mechanism in response to AID-induced DNA damage in non-B cells turns an otherwise negative result into a very interesting and biologically important study.

Minor points:

1. p.6, the abbreviation DSS is not defined.

2. p.6, Fig. 1D is not referred to in the text.

3. The axes in Figure 1 should be better labeled, for example, AID mRNA (relative expression), and Fig. 1D Y axis (frequency of lesions?).

4. Fig. 4B,D and Fig. 5E need stats. If the difference is not significant, this should be stated.

5. Fig. 5A, what kind of foci are being counted? Fig. 5C is very dark and doesn't show up well. Perhaps a higher magnification would help. The same is true for Fig. 3D.

6. p.7, please indicate the timeframe of adenocarcinoma development in other mouse models.

7. p.9, please state the significance of Ki67 staining, that it indicates cells in cycle.

8. p.11, bottom. The authors state that their findings "reinforce a well established link between AID activity and transcription of its target genes". The authors find no mutations in trp53 in their system. Is trp53 transcribed in these cells? Non-transcribed genes can be AID targets due to non-B DNA structure, for example, and so this statement should either be removed, because the data set is too limited, or modified merely to indicate that the genes they found mutated are, in fact transcribed.

Referee #3 (Remarks):

"AID-expressing epithelium is protected from oncogenic transformation by an NKG2D surveillance pathway"

Summary: AID deregulation in epithelial cells (at levels equivalent to those found in activated B cells) does not promote neoplastic transformation. Instead, it promotes the expression of NKG2D ligands, which would promote the clearance of precancerous cells.

The results are original and the goal of the study is potentially important since the consequences of AID deregulation have not been investigated in epithelial cells. However, the results are either negative (Tumor formation) or non-conclusive/over-interpreted (NKG2D/immune-surveillance). In general, the detection of few uncharacterized proliferating caspase-positive cells falls very short of a demonstration that there is tumor rejection upon AID deregulation.

Main points:

1. A general deficiency of the study is the poor documentation of the immune-istochemical analysis, a critical part of the results. In no case the (very few) cells positive for Ki-67, NKG2D or Caspase are AID-expressing epithelial cells.

2. The authors propose quantification of Ki67+ cells in tissues as a way to detect precancerous transformation. However, proliferation is not sinonimous of pre-malignancy or malignancy. it would

be important to quantify the number of Ki67+ cells among the AID+ epithelial cell pool. It is important to note that inflammatory infiltrates can be Ki67+.

3. It is unclear which region of the two Elastase genes did the authors screen for mutations. Are the mutations present in the first 2Kb from the TSS (as expected for AID activity) or other gene regions? The evidence of AID activity is very preliminary at best.

4. Gasser et al (Nature, 2005) reported that the level of DNA damage and subsequent NKG2D ligand induction were far higher and more represented in the cell population than those observed by the authors upon AID deregulation. They underscored that high levels of NKG2D ligands were important to promote immune recognition. Therefore, it is unclear how the relatively low levels of DNA damage (gamma-H2Ax) and NKG2D ligands in AID transgenic pancreas epithelium correlate with the obvious differences in immune response detected by the authors (for example, the presence of T cell clusters).

5. Along the same lines, Gasser et al showed that NKG2D ligand induction was dependent on ATM/ATR, and could therefore be reduced or prevented by exposure to ATM/ATR inhibitors, or ATM siRNA. It would be important to show caffeine of more specific ATM inhibitors would prevent the accumulation of T cells in AID overexpressing tissues.

6. There is a lack of consistency among data. Specifically, the information on DNA damage levels and mutational levels are given for >75wk old mice, but other features (e.g T cell infiltrates) are analyzed in younger mice (20wk old), for which DNA damage and mutational data are lacking.

1st Revision - authors' response 23 June 2015

Point-by-point Reply to Referees' comments

Referee #1 (Remarks)

The question whether inflammation-induced AID expression plays a role in carcinogenesis has not been addressed before and is of high relevance in the field. Here, the authors developed tissue specific overexpression models to determine if AID overexpression is sufficient to drive tumor development upon. The data show that AID-deficiency is not associated with a lower incidence of oncogenic lesions in a model of induced-colitis. At the same time tissue specific AID overexpression to levels typically observed in B cells was not sufficient to drive tumor development in colon and pancreas independent of the accumulation of mutations and presence of double-strand breaks. Using the pancreas model, the authors provide mechanistic evidence that activation of the DNA damage response may lead to NKG2D ligand expression and recruitment of cytotoxic T-cells to mediate cell death.

The manuscript is technically very solid in that the experiments are well controlled. The use of tissue-specific models advances significantly over the current literature and explains differences compared to previous reports of transgenic mice. The manuscript is well written and the conclusions are supported be the findings, which are novel and demonstrate that more thorough experiments are needed to define the importance of AID in epithelial tumor development.

We are extremely grateful to the reviewer for his/her enthusiastic assessment of our manuscript.

Referee #2

(Comments on Novelty/Model System)

Although statistical analysis is missing in some places, indicated in comments to the author, the experiments utilize state of the art technology and are extremely well performed. A novel system is used to express AID conditionally in two different tissues, so that the question of AID-induced tumorogenesis can be properly addressed.

(Remarks)

Activation induced cytidine deaminase (AID) induces DNA mutations and breaks in B cells that promote immunoglobulin class switch recombination and somatic hypermutation, and its activities are also strongly linked to lymphomogenesis. AID expression was initially thought to be restricted to B cells, but is now found in a broader array of tissues. The biological significance and consequences of AID expression in non-B cells is not known. Perez-Garcia et al ask whether the mutagenic activities of AID do, in fact, lead to tumorogenesis in non-B cells, using a model where AID expression is known to be induced during inflammation by TNFα in endothelial cells. This model is appropriate and of interest considering the known association between the inflammatory milieu and tumorogenesis in epithelial cells, and previous reports that suggest an association between AID expression and colorectal adenocarcinoma. The manuscript is very well written, interesting, and will appeal to a broad audience.

The authors confirm that endogenous AID is induced by inflammatory stimuli when pancreatic explants are treated with TNFα. They then use an established model of DSS-induced colitis associated cancer, but find no difference in pathology between AID-deficient and AID-heterozygous mice, and conclude that endogenous AID levels do not contribute significantly to colorectal adenocarcinoma. They go on to express AID at higher levels, similar to those seen in B cells, conditionally, under the control of promoters that direct expression specifically in colon or pancreas. This is in contrast to previous studies that have asked this question using transgenic mouse models where AID is broadly expressed and could induce secondary effects. In both tissues, they find that AID expression neither promotes tumor formation, nor impacts lifespan. Although this is essentially a negative result, it represents an important answer to a biologically important question, and the experiments were very well done so as to generate a meaningful result. Importantly, the authors go on to show that AID does in fact generate DNA lesions in these cells, and they identify a DNA damage response pathway induced in these cells that results in apoptotic death of damaged cells. Identification of this protective mechanism in response to AID-induced DNA damage in non-B cells turns an otherwise negative result into a very interesting and biologically important study.

The authors appreciate very much the insightful reading and positive opinion on the manuscript by Referee 2. All minor points and clarifications have now been addressed in the manuscript, as detailed below.

Minor points:

1. p.6, the abbreviation DSS is not defined.

Dextran sulfate sodium (DSS) is now defined (p6)

2. p.6, Fig. 1D is not referred to in the text.

Fig. 1D is now called on page 6.

3. The axes in Figure 1 should be better labeled, for example, AID mRNA (relative expression), and Fig. 1D Y axis (frequency of lesions?).

We have followed the reviewer suggestions and relabeled Figure 1A, B, C and D Y axes.

4. Fig. 4B,D and Fig. 5E need stats. If the difference is not significant, this should be stated.

The differences are not significant and this is now clarified in the text by saying:

"Acinar cells from R26AID$^{+/KI}$p48-CRE$^{+/KI}$ mice expressed higher levels of Raee than their control littermates, although the difference was not statistically significant (Fig. 4B)."

"We found that primary explants from R26AID$^{+/KI}$p48-CRE$^{+/KI}$ tended to be more sensitive to NK-mediated killing than R26AID$^{+/+}$p48-CRE$^{+/KI}$ littermate controls (Figure 4D)"

"R26AID$^{+/KI}$p48-CRE$^{+/KI}$ pancreas contained cells undergoing apoptotic cell death, detected by caspase3 immunohistochemistry (Fig. 5E, p=0.054)"

5. Fig. 5A, what kind of foci are being counted? Fig. 5C is very dark and doesn't show up well. Perhaps a higher magnification would help. The same is true for Fig. 3D.

The immune infiltrates were quantified by counting the number of groups of immune cells, whose size varied between a few dozens to possibly several thousand cells. We agree that "foci" is not a good term to define this, and have relabeled the 5A axis as "Immune clusters/mm^2"

We have now improved the quality of the microscope images shown in 5C and 3D.

6. p.7, please indicate the timeframe of adenocarcinoma development in other mouse models.

We thank the reviewer for bringing up this point. There are multiple mouse models for pancreatic ductal adenocarcinoma (PDAC) and colorectal adenocarcinoma. When expression of mutant KRas is activated in all cells of the pancreas early during pancreatic development (e9.5-e10) 65% of the mice have developed PDAC by one year (Martinelli et al. Gut 2014 Jan 16). Inactivation of tumor suppressors such as p16 and Trp53 - or activation of a mutant Trp53 allele concomitantly with mutant KRas - dramatically accelerates tumor development. In other mouse models of pancreatic cancer, such as the Ela-myc model, all mice have developed tumors by 5 months of age (Aguilar et al, 2004 Am J Pathol 165:1129). Regarding intestinal cancer, Min mice carrying one Apc mutant allele develop multiple intestinal adenomas by 3 months of age and most mice die before 6 months of age (Fodde and Smits 2001Trends in Mol Med 7:369). Inactivation of both Apc alleles in intestinal stem cells is also associated with widespread development of intestinal tumors and early death with high penetrance (Fodde and Smits 2001Trends in Mol Med 7:369).

Therefore the timeframe of adenocarcinoma onset in most cases ranges from few months to around a year. This is now stated in the manuscript on p7.

"The onset of pancreatic and colorectal adenocarcinoma in a variety of mouse models ranges from 5-6 months to 1-1.5 years"

7. p.9, please state the significance of Ki67 staining, that it indicates cells in cycle.

We apologize for this oversight; this is now clarified in the text.

8. p.11, bottom. The authors state that their findings "reinforce a well established link between AID activity and transcription of its target genes". The authors find no mutations in trp53 in their system. Is trp53 transcribed in these cells? Non-transcribed genes can be AID targets due to non-B DNA structure, for example, and so this statement should either be removed, because the data set is too limited, or modified merely to indicate that the genes they found mutated are, in fact transcribed.

The reviewer is right that this was an overstatement. We have rephrased the sentence as follows:

"AID mutagenic activity was detected in two highly expressed pancreatic genes (Ela1 and Ela2), which is expected from the well-established link between AID activity and transcription of its target genes"

Referee #3 (Remarks)

"AID-expressing epithelium is protected from oncogenic transformation by an NKG2D surveillance pathway"

Summary: AID deregulation in epithelial cells (at levels equivalent to those found in activated B cells) does not promote neoplastic transformation. Instead, it promotes the expression of NKG2D ligands, which would promote the clearance of precancerous cells.

The results are original and the goal of the study is potentially important since the consequences of

AID deregulation have not been investigated in epithelial cells. However, the results are either negative (Tumor formation) or non-conclusive/over-interpreted (NKG2D/immune-surveillance). In general, the detection of few uncharacterized proliferating caspase-positive cells falls very short of a demonstration that there is tumor rejection upon AID deregulation.

We are grateful to Referee #3 for agreeing to review our manuscript and for considering our results original and our study potentially important. We believe his/her comments and suggestions have helped us to significantly improve the quality of our manuscript. We have addressed his/her specific concerns as follows:

Main points:

1. A general deficiency of the study is the poor documentation of the immune-istochemical analysis, a critical part of the results. In no case the (very few) cells positive for Ki-67, NKG2D or Caspase are AID-expressing epithelial cells.

We agree with the Reviewer that this point was not sufficiently clarified. The immunohistochemistry was performed on pancreatic sections. We have now included higher magnification micrographs that unmistakably show that Ki67+ and Caspase+ stainings correspond to epithelial cells (Figure 4A, right panels and Figure 5E, right panels). Only epithelial cells were quantified for both stainings. In addition, we have performed new immunofluorescence stainings to combine Ki67 detection with an epithelial cell marker, cytokeratin-8 (CK8) (Figure 4B), and consistently find Ki67 and CK8 co-staining in the very same cells. These new data show that the increase rate of proliferating and apoptotic cells is accounted for by epithelial cells only, which greatly adds to the characterization of these events. Regarding detection of NKG2D ligand expression, FACS analysis was performed specifically on epithelial cells from pancreatic explants, which is now explicitly stated in the text. In the case of NKG2D detection by ddPCR, given that whole extracts were analyzed, we cannot rule out amplification from immune cells; however, please note that the result is consistent with the FACS analysis.

2. The authors propose quantification of Ki67+ cells in tissues as a way to detect precancerous transformation. However, proliferation is not sinonimous of pre-malignancy or malignancy. it would be important to quantify the number of Ki67+ cells among the AID+ epithelial cell pool. It is important to note that inflammatory infiltrates can be Ki67+.

The reviewer is absolutely right that Ki67 expression alone is not proof of malignancy or pre-malignancy. Therefore we have rephrased this sentence to restrict the scope of this finding, like this:

"pancreas from aged R26AID$^{+/KI}$p48-CRE$^{+/KI}$ mice contained more proliferating cells, as assessed by Ki67 staining, than control pancreas (Figure 4A), indicating that pancreatic AID expression leads to an abnormal rate of cell division"

As discussed in Point 1 above, we now provide a better characterization of Ki67+ cells, and show that AID-expressing epithelial cells have an increased rate of proliferation. Firstly, by morphological criteria alone (used for accurate quantification) (Figure 4 A, micrograps on the right) and secondly by CK8 expression, as measured by immunofluorescence (Figure 4B).

3. It is unclear which region of the two Elastase genes did the authors screen for mutations. Are the mutations present in the first 2Kb from the TSS (as expected for AID activity) or other gene regions? The evidence of AID activity is very preliminary at best.

The reviewer is indeed correct in that AID mutations are expected to occur preferentially at the first 2kb downstream of the TSS, a feature that is possibly related with the seemingly topological requirement of transcription for AID activity. This is why we measured AID-induced mutations by performing PCR-seq on highly transcribed genes at regions that cover roughly 800-900bp downstream of the TSS (Ela1: -22 to +840, chr15 100674400-100675240; Ela2a: -66 to +896, chr 4 141814918-141815814), and comparing mutation frequencies between AID-expressing pancreas and control littermates. This is now specifically mentioned in the text. Detecting AID activity is extremely challenging, and this kind of experiment, combined with Sanger validation, even if

limited, is considered the gold standard approach.

4. Gasser et al (Nature, 2005) reported that the level of DNA damage and subsequent NKG2D ligand induction were far higher and more represented in the cell population than those observed by the authors upon AID deregulation. They underscored that high levels of NKG2D ligands were important to promote immune recognition. Therefore, it is unclear how the relatively low levels of DNA damage (gamma-H2Ax) and NKG2D ligands in AID transgenic pancreas epithelium correlate with the obvious differences in immune response detected by the authors (for example, the presence of T cell clusters).

The reviewer raises the interesting question of the amount of DNA damage and the levels of NKGD2 ligand expression required to promote immune infiltration/effector function. Unfortunately, we are at this point unable to compare our data with those reported by Gasser et al (2005), because the model systems are too disparate to draw meaningful conclusions. The study by Gasser at al studied the expression of NKG2D ligands with a different reagent (tetrameric NKG2D) mostly in vitro, as opposed to using a mouse model. They neither assessed the presence of gamma-H2AX foci as a measurement of DNA damage, nor did they address immune cell recruitment, but rather characterized the DNA damage response leading to NKG2D ligand expression in vitro. In spite of this limitation, we agree that this is an interesting issue that deserves further attention with adequate experimental systems.

5. Along the same lines, Gasser et al showed that NKG2D ligand induction was dependent on ATM/ATR, and could therefore be reduced or prevented by exposure to ATM/ATR inhibitors, or ATM siRNA. It would be important to show caffeine of more specific ATM inhibitors would prevent the accumulation of T cells in AID overexpressing tissues.

This is again a valuable suggestion that would strengthen the idea of a DNA damage response downstream of the detected DNA damage. Unfortunately, this issue cannot be addressed with ATM/ATR inhibitors or siRNA, because assessing immune infiltration would require specific deletion in vivo, for instance with a conditional ATM knock-out model that should be bred to p48-Cre-Ki and ROSA-AIDKi alleles. Given that ATM/ATR activation by DNA double strand breaks (detected as gamma-H2AX foci in our study) is well-established, we believe that this type of experiment probably falls beyond the scope or our work.

6. There is a lack of consistency among data. Specifically, the information on DNA damage levels and mutational levels are given for >75wk old mice, but other features (e.g T cell infiltrates) are analyzed in younger mice (20wk old), for which DNA damage and mutational data are lacking.

We apologize for the omissions that have made this issue confusing. Immune infiltrate analysis (Fig 5A, B), as well as Ki67 and Caspase detection by immunohistochemistry (Fig. 4A and 5E) and NKG2D ligand and TNF-alpha expression (Fig. 4D and 5D) were performed in aged mice (>= 75 weeks old). However, immune infiltrates (Fig. 5C) and Ki67 expression (Fig. 4B) were also detected in younger animals (i.e. 20 weeks old). Mutation analysis (Fig 3A and B) was done in pancreas from 20 week-old mice. Finally, all experiments requiring pancreas explants (DNA damage in Fig. 3C and D; FACS analysis of NKG2D ligands in Fig. 4C and Killing assay in Fig. 4E) were necessarily done in even younger animals (8 weeks old) to enable explant viability. In summary, although many of the “effector” features were first analyzed and detected at end-point time, when presumably tumors might have arisen, our data indicate that these events are detectable much earlier; specifically, this is true for the presence of mutations and of DNA damage. Age information is now included in the corresponding Figure Legends and Materials and Methods sections.

2nd Editorial Decision 08 July 2015

Thank you for the submission of your revised manuscript to EMBO Molecular Medicine. We have now received the enclosed reports from the referees who were asked to re-assess it.

As you will see, while reviewer #2 is now supportive, reviewer #3 still raised issues that s/he doesn't feel were properly addressed in the revision. As the issues were rather of a technical nature, I asked referee 2 to cross-comment. This referee believes that technically referee 3 is right. However, the

provision of GFP expression, mRNA and increased damage assayed by several means, all corroborate and point to AID-dependent damage, which answers referee 3 point 1. Regarding point 2, while indeed the mutation data is low, it remains in the ballpark of other off-target mutation rates. Therefore, we do request that you reply point-by-point to referee 3 comments in writing and make the limitations clearer in the main text.

Please submit your revised manuscript within two weeks. I look forward to seeing a revised form of your manuscript as soon as possible.

***** Reviewer's comments *****

Referee #2 (Remarks):

The revised manuscript is very much improved. Quality of the microscopic figures is now excellent. All reviewers' concerns appear to have been addressed.

Referee #3 (Remarks):

I cannot consider the revision as satisfactorily addressing some of my most important concerns. Specifically:

1. Although additional IHC and IF documentation is added, none of them documents the expression of Ki67, NKG2D, gamma-H2AX or Caspase in AID-expressing cells, i.e. by co-staining any of the above markers with AID. AID Abs are available commercially and have been used in IHC and IF analysis in numerous papers.Indeed, there is no evidence that the AID protein is expressed in the entire paper (documentation of RNA expression is not sufficient).

2. The evidence of AID mutational activity remains poorly documented. DNA is extracted from "pancreas" (a very complex tissue) and analyzed for mutations. The number detected is extremely low (the authors may be comparing their overall tissue-frequency with the single-cell or single-allele frequencies reported in in the literature) and of unclear significance considering the uncertainty on the fraction of AID expressing cells (see point 1). In any case, the type and distribution of the mutations is not documented.

2nd Revision - authors' response 16 July 2015

Point-by-point Reply to Referees' comments

Referee #2 (Remarks):

The revised manuscript is very much improved. Quality of the microscopic figures is now excellent. All reviewers' concerns appear to have been addressed.

We are very grateful to Referee #2 for helping us improve the clarity and quality of our manuscript.

Referee #3 (Remarks):

1. Although additional IHC and IF documentation is added, none of them documents the expression of Ki67, NKG2D, gamma-H2AX or Caspase in AID-expressing cells, i.e. by co-staining any of the above markers with AID. AID Abs are available commercially and have been used in IHC and IF analysis in numerous papers. Indeed, there is no evidence that the AID protein is expressed in the entire paper (documentation of RNA expression is not sufficient).

We agree with the reviewer that co-staining of any of Ki67, NKG2D ligand, gamma-H2AX or Caspase with AID, would have been optimal. Unfortunately, immunohistochemical or immunofluorescence analysis of AID expression on tissue sections, while very often reported for

human samples, is truly challenging for mouse samples, and indeed constitutes one critical technical issue in the field. Therefore, this has been technically impossible. However, we believe that the findings that GFP is expressed in epithelial cells, that AID mRNA is detected, and that the R26AIDki/+ mice but not R26AID+/+ controls show these phenotypes, is evidence of AID-dependency. Regarding the presence of AID protein, we provide proof here by western blot of pancreatic extracts that hope will satisfy the reviewer on this point (R#3 Fig.1).

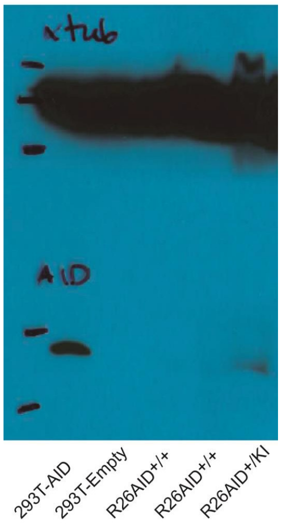

R#3 Figure 1. AID protein is detected in pancreas from R26AID$^{+/KI}$ p48CRE $^{+/KI}$ mice. Whole pancreatic lysates from 8 week-old R26AID$^{+/+}$ p48CRE $^{+/KI}$ and R26AID$^{+/KI}$ p48CRE $^{+/KI}$ mice were immunoblotted to detect AID protein (anti-AID mAID-2, e-Bioscience 14-5959). Whole cell lysates from 293T cells transfected with AID (293T-AID) or an empty vector (293T-Empty) were used as controls. Immunoblot with anti-tubulin is shown as control.

2. The evidence of AID mutational activity remains poorly documented. DNA is extracted from "pancreas" (a very complex tissue) and analyzed for mutations. The number detected is extremely low (the authors may be comparing their overall tissue-frequency with the single-cell or single-allele frequencies reported in the literature) and of unclear significance considering the uncertainty on the fraction of AID expressing cells (see point 1). In any case, the type and distribution of the mutations is not documented.

The reviewer is right that pancreas is a complex tissue and therefore, that our mutation analysis is done on a heterogenous sample. However, a very big fraction of the cells analyzed are AID+, as expected by the genetic system used (p48Creki/+) and as indicated by the pattern of GFP expression. Still, we agree that a fraction of AID- cells are included in the analysis that may "dilute" the total mutation frequency. In any case, the frequencies obtained in this study are in the same frequency range as those reported for other off-target genes (see, for instance, Liu et al., Nature 2008, 451:841, cited in the reference section). Regarding this, it is important to keep in mind that in our model, mutations are non-clonal -given that they are not positively selected during neoplasia development, which does not occur. This is in sharp contrast with numerous studies performed on lymphoma or other cancer situations, where mutation frequency can be expectedly higher. Finally, mutations are shown to accumulate preferentially at AID hotspots (Fig. 3A and B), a further evidence of AID dependent mutagenesis.
